# Effect of *Hippophae rhamnoides* Extract Addition on the Quality and Safety of Traditional Kazakh Chunked Delicacy “Jaya”

**DOI:** 10.3390/foods14213698

**Published:** 2025-10-30

**Authors:** Mariam K. Alimardanova, Sholpan A. Abzhanova, Aktoty N. Kurmanali, Nikolay D. Kolev, Anastasya D. Yankova-Nikolova, Nevena N. Nacheva-Dimitrova, Desislava B. Vlahova-Vangelova, Dessislav K. Balev, Stefan G. Dragoev

**Affiliations:** 1 Department of Food Technology, Faculty of Food Technology, Almaty Technological University, 100 Tole bi str., 050012 Almaty, Kazakhstan; alimardan.m.atu4@mail.ru; 2 Department of Food Biotechnology, Faculty of Food Technology, Almaty Technological University, 100 Tole bi str., 050012 Almaty, Kazakhstan; tolkyn_07.08@mail.ru; 3 Department of Meat and Fish Technology, Technological Faculty, University of Food Technologies, 26 Maritza Blvd., 4020 Plovdiv, Bulgaria; n_kolev@uft-plovdiv.bg (N.D.K.); a.yankova@gmail.com (A.D.Y.-N.); n_nacheva@uft-plovdiv.bg (N.N.N.-D.); or desislava_vangelova@abv.bg (D.B.V.-V.);; 4 Assembly of Academicians and Corresponding Members, Bulgarian Academy of Sciences, 1 Fifteenth of November str., 1040 Sofia, Bulgaria

**Keywords:** horse chunked meat, smoked ham, sea buckthorn, functional meat product, bioactive substances, natural extracts, extract proportion

## Abstract

The present study aimed to evaluate the effect of incorporating 3.0% powdered water–ethanol extract from dried sea buckthorn pomace on the quality and safety of the traditional Kazakh chunked delicacy Jaya. The optimal extraction conditions were established as 70% ethanol at an ethanol-to-dry sea buckthorn pomace ratio of 1:5, yielding the highest sensory-evaluated consumer preference. A two-way ANOVA was employed to assess the influence of extract supplementation and refrigerated storage for 30 days at 0–4 °C on instrumental colour, pH, acid and peroxide values, TBARS, texture profile, total phenolic content (TPC), antioxidant activity, and microbiological status of the finished products. A statistically significant (*p* < 0.05) increase in total phenolic content (18.6%), radical scavenging activity against DPPH (9.6%), and ferric reducing antioxidant power (FRAP) (14.9%) was observed. The addition of 3.0% sea buckthorn extract exerted a moderate effect in reducing oxidative processes in Jaya. However, decreases in pH (from 6.10 to 5.93), discolouration of the cut surface, and changes in the texture profile were noted. The incorporation of dried sea buckthorn extract may thus be effectively applied in the production of the traditional Kazakh chunked delicacy Jaya, contributing to enhanced oxidative stability, although it may not improve pH, colour, or texture characteristics. Due to limitations of the present study related to the addition of only one extract and a limited methodological panel, the need to conduct an additional series of studies in the future is justified, in order to establish the effect of adding lower levels (<3%) of the inclusion of the powdered extract of dried sea buckthorn pomace on the quality and stability of the product.

## 1. Introduction

Traditional Kazakh cuisine is distinguished by a variety of meat-based products [1]. Among these, one of the most widely consumed and culturally significant is “Jaya,” which holds both historical and cultural value [2]. “Jaya” is a delicacy consisting of cooked, smoked meat, typically prepared during the winter months. The main ingredients for this product include beef or horse meat, salt, sugar, and various spices [1,2]. Despite its recognition for its distinctive flavour and traditional preparation methods, “Jaya” is prone to lipid and pigment oxidation [3]. Such oxidative degradation, along with microbiological spoilage, not only affects the sensory properties of the product but also leads to a loss of nutritional value and a reduction in shelf life [4].

In recent years, numerous strategies aimed at enhancing the functional properties of meat and meat products have been discussed within the scientific community [5]. One such approach involves the incorporation of sea buckthorn preparations, which are rich in natural polyphenols known for their potent antioxidant properties. For instance, sea buckthorn oil has been suggested as a means to improve the sensory and physicochemical qualities of functional meat products [6]. Furthermore, products derived from sea buckthorn processing have been utilised in the creation of biologically active food emulsions [7].

Sea buckthorn fruits (*Hippophae rhamnoides* L.) represent a valuable functional food resource [8], containing a broad spectrum of lipophilic and hydrophilic bioactive compounds [9,10]. These compounds have found extensive use, both as functional additives in the food industry [11,12] and as a potential source of biologically active substances [13] with therapeutic applications [14,15,16]. The therapeutic potential of *Hippophae rhamnoides* L. is well-documented, with known anti-inflammatory [17,18,19], immunostimulating [20], chemoprophylactic and therapeutic properties [21], as well as antiulcerogenic [22], antihypertensive [23], and antiosteoporotic effects [24]. Accordingly, a variety of biologically active compounds derived from sea buckthorn have been incorporated into food products designed for the prevention of various diseases [25].

In meat products, sea buckthorn preparations are primarily utilised for their high antioxidant [26] and antimicrobial properties [27,28]. Their in vitro antioxidant effects are attributed to their notable ability to scavenge free radicals [29,30]. For instance, ground sea buckthorn fruits have been added to pork sausages [31], fruit juice has been incorporated into beef burgers [32], and dried fruit powder has been used in a functional horsemeat delicacy [33].

Additionally, an edible potato starch film, incorporating various concentrations of a water–ethanol extract from sea buckthorn pomace, has been employed to extend the shelf life of beef jerky and super-chilled beef (−1.3 °C) [34,35]. The use of different solvents for extracting various components of sea buckthorn has also been explored in the literature [36,37,38,39], and extraction protocols aimed at obtaining phenolic-rich antioxidants from sea buckthorn have been optimised using the response surface methodology [40].

Despite the wealth of research on sea buckthorn, there appear to be no studies specifically addressing the impact of powdered 70% ethanol extracts of defatted by-products (i.e., dry-pressed pomace of *Hippophae rhamnoides*) on cooked-smoked horsemeat delicacies. Therefore, the objective of the present study was to evaluate the influence of the addition of powdered ethanol extract from defatted sea buckthorn pomace on the quality and safety of the traditional Kazakh delicacy, Jaya.

## 2. Materials and Methods

### 2.1. Materials

*Horse meat.* Chilled, deboned horse rump and fat were obtained from the slaughterhouse of Pervomaiskiye Delikatesy Ltd. (Kasyksky village, Kordaysky district, Kazakhstan) 48 h post mortem.

**Figure 1:**
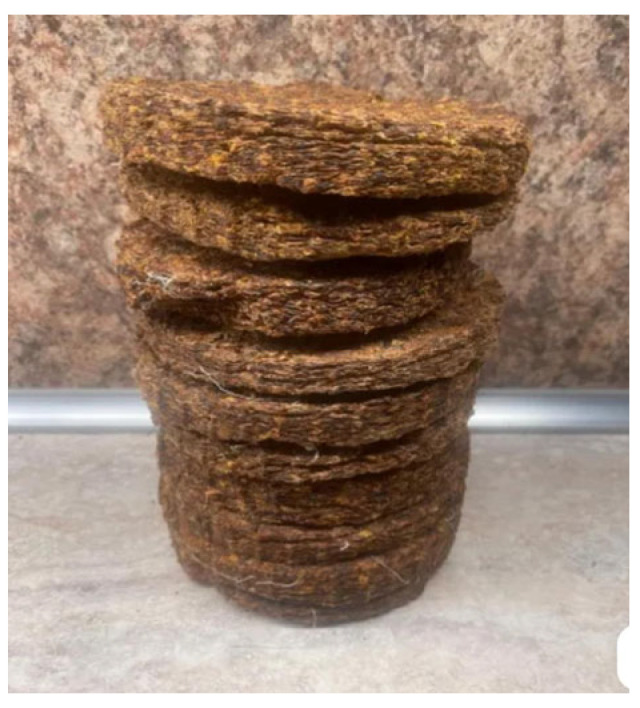
Defatted sea buckthorn pomace.

**Figure 2:**
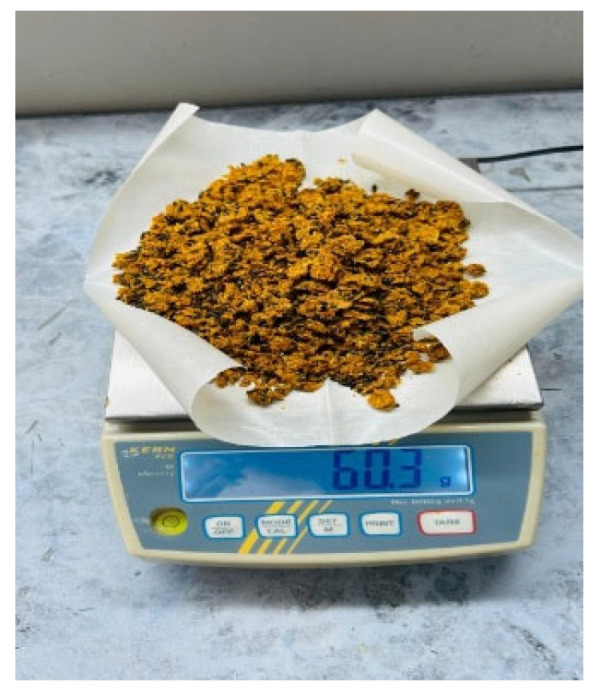
Crushed dried sea buckthorn pomace obtained after oil extraction.

*Defatted sea buckthorn pomace*, a by-product of juice processing (Figure 1), was procured from Yuantai Organic Ltd. (Tiangu, Yanta District, Xi’an, Shaanxi, China). The material was dried at 50 °C for 18 h to reduce the moisture content while preserving the structural integrity. The dried pomace was subsequently ground into a fine powder (≤3 mm) (Figure 2).

*All reagents and standards* were an analytical grade and were purchased from Laborpharma Ltd. (Almaty, Kazakhstan) or Labhimprom Ltd. (Almaty, Kazakhstan).

### 2.2. Experimental Design

#### 2.2.1. Ultrasound-Assisted Ethanol Extraction of Crushed Dry Defatted Sea Buckthorn Pomace

**Figure 3:**
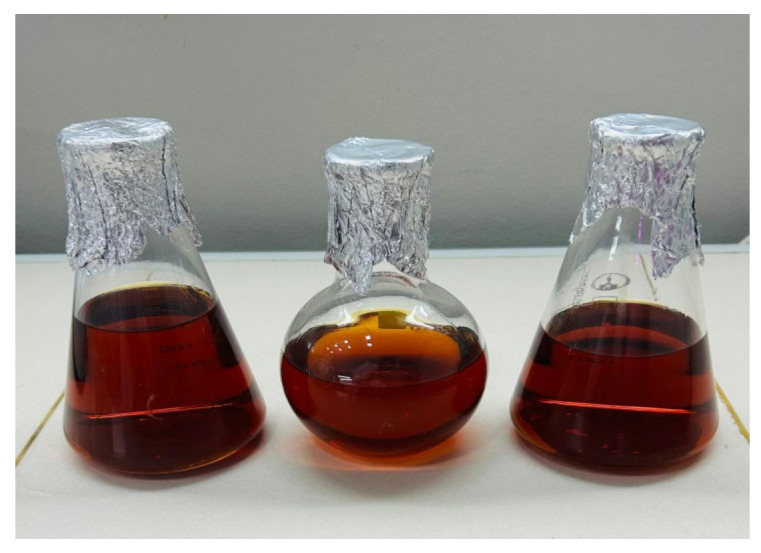
Liquid extract from dried sea buckthorn pomace after filtration.

**Figure 4:**
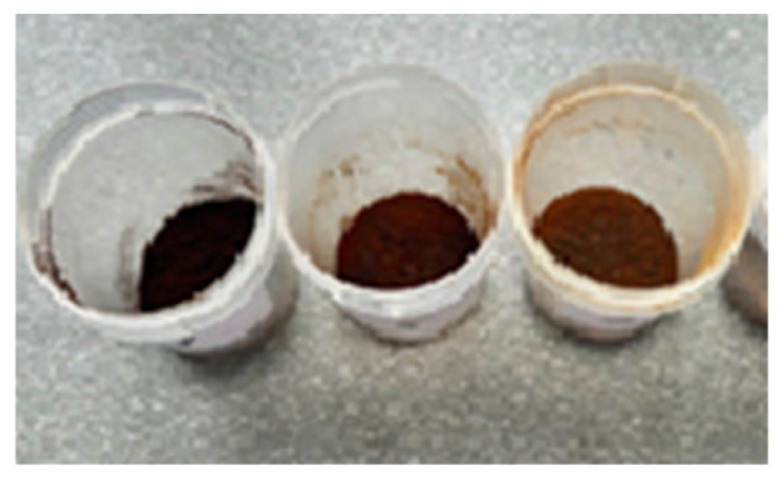
Dry extracts obtained from dried sea buckthorn pomace.

Ultrasonic ethanol extraction was performed using three ethanol concentrations—50%, 70%, and 90%—at dry flake-to-solvent ratios of 1:5, 1:10, and 1:15 (*w*/*v*). The extraction was conducted in a TTC Sapphire 4 L ultrasonic bath (JSC Sapphire, Moscow, Russia) operating at 35 kHz and 150 W, at 60 °C for 30 min. The resulting extracts (Figure 3) were filtered and subsequently centrifuged using a PE-6900 centrifuge (GEO-NDT LLC, Moscow, Russia) at 4000 min^−1^ for phase separation. Ethanol was then removed via rotary evaporation using an IKA RV 5 rotary evaporator (IKA–Werke GmbH & Co., Staufen im Breisgau, Germany) at a bath temperature of 60 °C under an absolute pressure of 5 kPa. The concentrated extracts were dried at 40–50 °C for 24–36 h to obtain a powdered form. The powdered sea buckthorn extracts (Figure 4) were reconstituted in distilled water and homogeneously mixed with nitrite salt and spices. The mixture was then moulded and air-dried according to traditional methods of Jaya production.

Preliminary technological experiments (Table 1) indicated that the highest extraction yield (18%) was achieved using 70% ethanol at a solvent-to-material ratio of 1:5. This result demonstrates the high efficiency of 70% ethanol as an extractant capable of solubilising both polar and moderately non-polar compounds, such as phenolic acids and flavonoids. Increasing either the solvent volume or the ethanol concentration beyond these levels resulted in reduced yields, likely due to dilution effects and decreased solubility of specific constituents.

The extract obtained with 50% ethanol exhibited a dry powder consistency, orange colour, light grassy aroma, and contained approximately 8% fatty acids.

The extract produced with 70% ethanol had a dark orange colour, characteristic sea buckthorn aroma, and 12% fatty acid content. It was characterised by a pH of 3.88 and a total phenolic content (TPC), determined by the Folin–Ciocalteu method, of 42 mg GAE/g of dry extract. The extract prepared using 90% ethanol displayed an oily powder consistency, brown colour, slightly oily odour, and 15% fatty acid content.

Based on these findings, the optimal extraction conditions were determined to be 70% ethanol at a solvent-to-raw-material ratio of 1:5. Consequently, this extract type was selected for further stages of the study. The decision to employ 70% ethanol extraction of defatted sea buckthorn flakes was supported not only by the preliminary results but also by literature evidence. Korekar et al. [37] reported that 70% ethanol yielded extracts of *Hippophae rhamnoides* L. (sea buckthorn) fruit pulp, seeds, leaves, and stem bark with the highest antioxidant capacity and total phenolic content. Furthermore, the adoption of ultrasound-assisted extraction in the present study was informed by the findings of Sharma et al. [39], who demonstrated that ultrasound and microwave-assisted methods offer superior efficiency compared with Soxhlet extraction and maceration.

#### 2.2.2. Preparation and Sensory Evaluation of Jaya Samples

**Figure 5:**
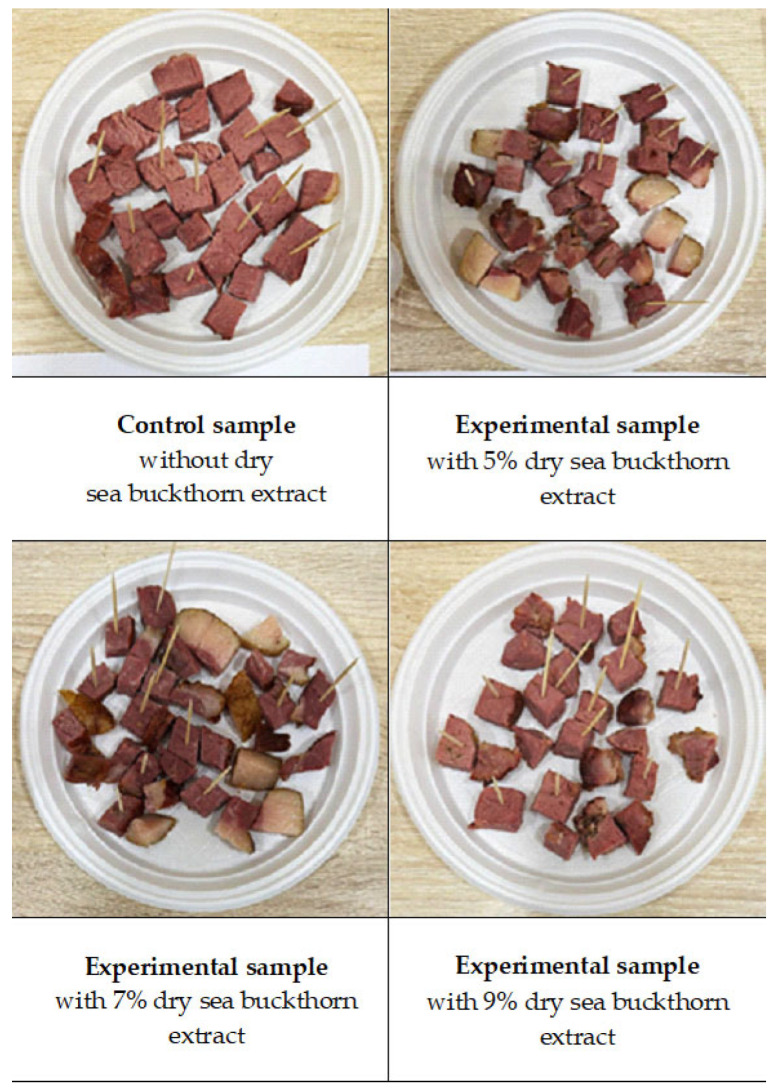
Effect of the addition of 5, 7 and 9% sea buckthorn extract on the sensory evaluated consumer preference of traditional Kazakh chunked delicacy “Jaya”.

Four Jaya samples were prepared: a control (without extract) and three experimental formulations incorporating 5%, 7%, and 9% sea buckthorn extract, respectively (Figure 5). The samples were subjected to sensory evaluation for consumer preference. A trained panel of ten assessors participated in three one-hour training sessions, during which reference samples representing varying intensities of colour (cross-sectional surface), aroma, flavour, texture, uniformity, and moisture were evaluated, following the guidelines of Meilgaard [41].

Consumer preference scores were obtained using a five-point hedonic scale (1 = strongly dislike; 5 = strongly like). Data were analysed by one-way analysis of variance (ANOVA), followed by Tukey’s post hoc test, with statistical significance set at *p* < 0.05. All analyses were performed using SPSS software, version 26.

The results indicated that the sample containing 5% sea buckthorn extract most closely resembled the control in appearance and texture. However, its flavour and aroma were more pronounced and not characteristic of the traditionally produced Jaya in Kazakhstan.

#### 2.2.3. Rationale for Determining the Optimal Level of Sea Buckthorn Extract

The findings of this preliminary investigation prompted us to review relevant studies by other researchers, although their experiments involved different types of meat products and various sea buckthorn preparations.

Guo et al. [34] recommended the incorporation of 2–4 wt% sea buckthorn flake extract into esterified potato starch films used for packaging dried beef, noting that a 6% addition adversely affected the product’s odour. In a subsequent study, Guo et al. [35] reported that a 3 wt% concentration of sea buckthorn flake extract was optimal for combined packaging of super-chilled beef with starch film stored at −1.3 °C.

Similarly, Wojtaszek et al. [33] proposed the addition of 1% sea buckthorn juice to stabilise the quality of beef burgers. It should be emphasised, however, that the total phenolic content of sea buckthorn juice (2758.9 ± 53.80 mg GAE/100 g) is several times higher than that of the dried preparation used in the present study.

Anchidin et al. [6] reached comparable conclusions, reporting high consumer acceptability of pork fillets injected with 1% and 3% sea buckthorn oil, while indicating that a 5% injection level was excessive and negatively affected sensory characteristics.

For cooked sausages, Saleida et al. [31] identified the addition of 3% ethanol extract of sea buckthorn fruits as the optimal concentration, whereas Kozhakhieva et al. [32] recommended producing a functional cooked-smoked horse meat delicacy (Jaya) by injecting a 20% brine containing 5 kg of powdered sea buckthorn fruit extract per 100 kg of meat (equivalent to approximately 1.0% in the finished product).

In light of these findings, we decided to proceed with further experiments employing a 70% brine solution containing 3 kg of defatted pulp flake extract per 100 kg of meat, corresponding to a final concentration of 2.1% in the finished Jaya product.

#### 2.2.4. Preparation of the Jaya Chunked Horse Meat Delicacy

The horse meat used in this study was delivered to the Educational and Scientific Centre of Meat Processing at the Almaty Technological University (ATU), Kazakhstan where the experimental production of the traditional chunked delicacy Jaya was carried out.

The chilled meat arrived at a temperature of 2 °C. Only lean muscle cuts, specifically from the rear rump (including m. semimembranosus, m. semitendinosus, and m. biceps femoris), free from visible fat, sinew, and connective tissue, were selected. This selection is critical to ensure a fine and tender texture in the final product, while also meeting hygienic food safety requirements. During preparation, layers of horse fat were placed between two pieces of muscle.

##### Brine Preparation and Injection

The formulation for 100 kg of Jaya consisted of 100,000 kg of horse meat and fat, injected with 70,000 kg of brine containing sea buckthorn extract. The brine (100 L total) was prepared by dissolving 3000 kg of sea buckthorn extract powder, 7000 kg of table salt, 1500 kg of nitrite curing salt (containing 0.6% NaNO_2_), and 0.250 kg of ground white pepper in 100 L of chilled water (2 °C). The brine was mechanically homogenised to ensure complete dissolution of the salts and uniform distribution of the extract.

A 70% brine injection (relative to the meat’s weight) was administered using a single-head injector (Günther PI 16, Günther Maschinenbau GmbH, Eppertshausen, Germany) equipped with 16 needles, operating at a pressure of 0.2 MPa.

##### Tumbling and Moulding

Following injection, the horse meat was vacuum-tumbled for 6 h at 2–4 °C. The tumbled pieces were then arranged in special aluminium moulds: a lower layer of horse rump meat, a middle layer of horse fat, and an upper layer of horse rump meat. The moulded Jaya pieces were removed from the forms, placed in a single layer on sausage-cart grills, and subjected to a three-phase heat treatment process.

##### Thermal Processing

Searing: The first stage involved searing at 80 °C for 90 min to remove surface moisture and prepare the product for further heat treatment.

Steaming: The second stage consisted of steaming at 80 °C for 70 min, until the internal temperature of the meat reached at least 73 °C.

Drying: The third stage involved hot-air drying at 60 °C for 10 min.

##### Smoking and Packaging

The cooked Jaya pieces were smoked for 20 min at 60 °C using beech wood chips, imparting the characteristic pronounced smoked aroma and flavour of traditional Jaya. The smoked products were then cooled within one hour to an internal temperature not exceeding 4 °C and hermetically vacuum-packed in multilayer barrier foil packaging.

**Figure 6:**
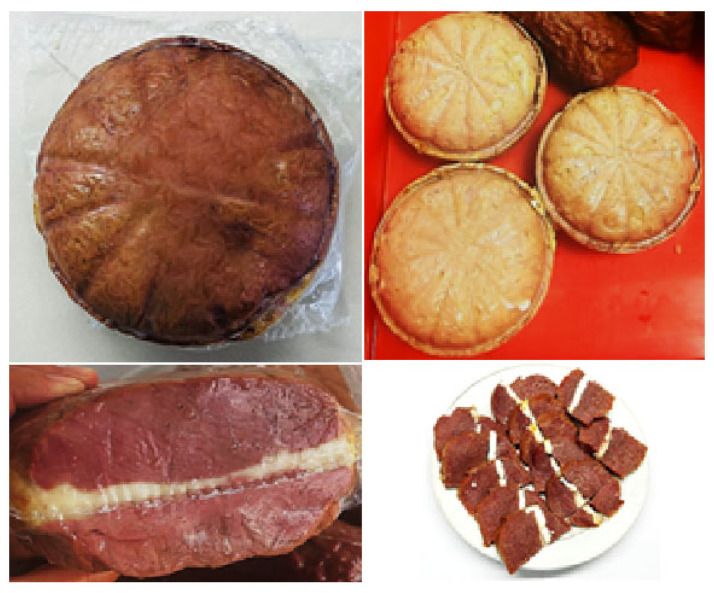
Traditional Kazakh chunked delicacy from horse meat “Jaya”.

After labelling, the packaged Jaya samples (Figure 6) were stored at 0–4 °C until further analysis.

#### 2.2.5. Determination of Quality Parameters of the Traditional Kazakh Chunked Horse Meat Delicacy Jaya

Two samples of the traditional Kazakh chunked horse meat delicacy *Jaya* were evaluated: a control sample (containing 0% sea buckthorn extract) and an experimental sample (containing 3% dried sea buckthorn extract). Both samples were stored for 30 days at 0–4 °C.

During the storage period, changes were assessed in instrumental colour parameters (L*, a*, b*), pH value, texture profile analysis (TPA), as well as hydrolytic and oxidative alterations in the lipid and protein fractions. In addition, total phenolic content (TPC), antioxidant activity, and microbiological quality were examined.

### 2.3. Methods

#### 2.3.1. Instrumental Colour

The instrumental colour parameters—lightness (L*), redness (a*), and yellowness (b*)—of the cross-sectional surface of the Jaya muscle pieces were determined using a Konica Minolta CR-410 colourimeter (Konica Minolta Holdings, Wayne, NJ, USA) following the procedure described by King et al. [42].

#### 2.3.2. pH Value

The pH value was measured using a Hanna pH meter, model HI99163 (Hanna Instruments, Smithfield, RI, USA), equipped with a meat contact sensor tip (type FC099). The instrument was calibrated before each use with certified buffer solutions of pH 4.04 and 6.86 [43].

#### 2.3.3. Texture Profile Analysis (TPA)

Samples were cut into cubes measuring 1 × 1 × 1 cm. Texture profile analysis was conducted using a TX-700 texture analyser equipped with a 25 kg load cell (Lamy Rheology, Champagne-au-Mont-d’Or, France), following the method of Kolev et al. [44].

#### 2.3.4. Extraction of Muscle Proteins

Muscle proteins were extracted following a modification of the method of Khan [45]. A 2.5 g meat sample was homogenised with 48.5 cm^3^ phosphate-buffered saline (49 mM Na_2_HPO_4_·7H_2_O, 4.5 mM NaH_2_PO_4_·H_2_O, KCl) to achieve an ionic strength of 0.55. The homogenate was conditioned for 12 h at 0–+4 °C and then centrifuged for 15 min at 1000× *g*.

#### 2.3.5. Hydrolytic and Oxidative Changes in Lipid Fraction

The acid value (AV) was determined according to ISO 660:2020 [46].

The peroxide value (POV) was measured using the method of Shantha and Decker [47] with a double-beam UV–Vis spectrophotometer, model M550 (CamSpec Ltd., Leeds, UK).

The 2-thiobarbituric acid reactive substances (TBARS) were quantified following the method of Botsoglou et al. [48], as modified by Moraru Manea et al. [49].

#### 2.3.6. Hydrolytic and Oxidative Changes in Protein Fraction

The free amine nitrogen (FAN) content was determined using a double-beam UV–Vis M550 spectrophotometer (CamSpec Ltd., Leeds, UK), according to the method of Vassilev et al. [50].

The concentration of carbonyl groups was measured following the method of Mercier et al. [51].

#### 2.3.7. Antioxidant Activity

The total phenolic content (TPC) was determined by the Folin–Ciocalteu method, following Vardakas et al. [52].

The radical scavenging activity against 1,1-diphenyl-2-picrylhydrazyl (DPPH) was evaluated according to the procedure of Brand-Williams et al. [53], as modified by Dinkova et al. [54].

The ferric reducing antioxidant power (FRAP) assay, assessing metal ion (Fe^3+^) chelating activity, was conducted using the modified method of Benzie and Strain [55], as described by Dinkova et al. [54].

#### 2.3.8. Sensory Analysis

Sensory evaluation of consumer preference for *Jaya* samples was conducted following the recommendations of Drake et al. [56].

#### 2.3.9. Microbiological Evaluation

Microbiological safety was assessed on days 1, 15, and 30 of storage at 0–+4 °C using four indicators:

Total aerobic plate count (TAPC)—determined according to GOST R 54354-2011 [57];

Moulds, yeasts, and spore-forming microorganisms—analysed by GOST 10444.15-94 [58];

Coliforms—detected in 1.0 g of product according to GOST 31747-2012 [59];

*Salmonella* spp.—examined in 25 g of product according to GOST 31659-2012 [60].

#### 2.3.10. Statistical Analysis

Data were statistically analysed using two-way ANOVA followed by Tukey’s post hoc test (*p* < 0.05) in GraphPad Prism software version is 10.6.1 (n = 6). The two experimental factors considered were storage time and the addition of dried sea buckthorn extract [61].

## 3. Results

### 3.1. Effect of Dry Sea Buckthorn Extract on the Instrumental Colour of the Traditional Kazakh Chunked Horse Meat Delicacy Jaya

The results of the instrumental colour measurements—lightness (L*), redness (a*), and yellowness (b*)—of *Jaya* samples during storage at 0–4 °C are presented in Table 2.

Both experimental factors, namely storage time and the addition of sea buckthorn extract, as well as their interaction, significantly (*p* < 0.05) influenced the instrumental colour of the chunked delicacy (Table 2). Lightness (L*) decreased significantly (*p* < 0.05) on the first day of refrigerated storage, but increased by 4.37 units after 30 days. In contrast, the red colour component (a*) decreased significantly on day 1, while the yellow component (b*) did not change significantly (*p* > 0.05) during the same period.

After 30 days of storage, the red component (a*) had decreased by approximately 23%, whereas the yellow component (b*) had increased by 29%. These results indicate that the addition of dry sea buckthorn extract and storage time have distinct and measurable effects on the colour attributes of *Jaya*, with implications for visual quality and consumer perception.

The results indicate that the addition of sea buckthorn extract caused significant (*p* < 0.05) discoloration of the *Jaya* cut surface, which appeared paler pink compared with the control sample.

### 3.2. Effect of Dry Sea Buckthorn Extract on the pH Value of the Traditional Kazakh Chunked Horse Meat Delicacy Jaya

**Figure 7:**
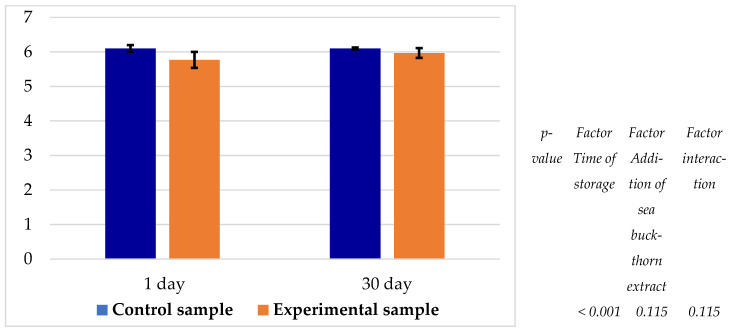
pH value of samples during storage at 0–4 °C.

It was observed that only the storage time significantly (*p* < 0.05) influenced the pH of the samples (Figure 7). A similar decreasing trend in pH was noted for both the control and experimental samples throughout the storage period. The pH of the experimental sample was consistently lower than that of the control by 0.13–0.33 units, ranging between 5.77 and 5.97.

### 3.3. Effect of Dry Sea Buckthorn Extract on the Texture Profile (TPA) of the Traditional Kazakh Chunked Horse Meat Delicacy Jaya

The texture profile analysis (TPA) parameters of the two Jaya samples during storage at 0–4 °C are presented in Table 3.

Storage time did not significantly (*p* > 0.05) affect hardness or cohesiveness, and the addition of sea buckthorn extract did not significantly influence resilience (*p* > 0.05). Furthermore, the interaction between storage time and extract addition had no significant effect (*p* > 0.05) on cohesiveness, springiness, chewiness, or resilience (Table 3).

The addition of 3% sea buckthorn extract, however, resulted in a significant (*p* < 0.05) reduction in hardness by a factor of 1.41–1.33. After 30 days of refrigerated storage, cohesiveness and resilience remained largely unchanged, whereas springiness, gumminess, and chewiness decreased significantly (*p* < 0.05) by 0.10, 23.38 N, and 21.24 N·cm, respectively (Table 3).

Overall, both the control and experimental samples exhibited a significant (*p* < 0.05) decline in springiness, gumminess, chewiness, and resilience over the 30-day storage period (Table 3), indicating that storage time exerts a measurable influence on the texture of Jaya.

### 3.4. Effect of Dry Sea Buckthorn Extract on Hydrolytic and Oxidative Changes in Lipid and Protein Fractions of the Traditional Kazakh Chunked Horse Meat Delicacy Jaya

The results of hydrolytic and oxidative changes in the lipid and protein fractions of the two *Jaya* samples during storage at 0–4 °C are presented in Table 4.

Both storage time and the addition of sea buckthorn extract significantly (*p* < 0.05) influenced the indicators of lipolytic changes, as measured by the acid value (AV). After 30 days of storage, AV increased significantly (*p* < 0.05) in both samples, by 15.2% in the control and by 40.7% in the experimental sample (Table 4).

The incorporation of 3% sea buckthorn extract caused a significant (*p* < 0.05) increase in AV at both time points, with an increase of 20% after 1 day and 40% after 30 days of storage (Table 4).

It should be noted, however, that the AV levels remained relatively low, ranging from 0.28 to 0.59 mg KOH/g, which is substantially below the 1.00 mg KOH/g threshold considered acceptable for high-quality meat products [40].

The addition of 3% sea buckthorn extract did not have a significant (*p* > 0.05) effect on primary lipid oxidation products, as measured by the peroxide value (POV); however, storage time significantly (*p* < 0.05) influenced POV, which increased by 0.34–0.44 meq O_2_/kg in both samples (Table 4). Similar to AV, POV levels remained relatively low, ranging from 0.11 to 0.55 meq O_2_/kg, well below the 1.00 meq O_2_/kg threshold considered acceptable for cooked meat products [43].

Storage time did not significantly (*p* > 0.05) affect secondary lipid oxidation products, measured as 2-thiobarbituric acid reactive substances (TBARS). In contrast, the addition of 3% sea buckthorn extract significantly (*p* < 0.05) reduced TBARS by 26.5–44.7% (Table 4). TBARS levels remained relatively low, ranging from 0.26 to 0.49 mg MDA/kg, which is below the 3.00 mg MDA/kg limit deemed acceptable for cooked meat products [43].

Neither storage time, the addition of sea buckthorn extract, nor their interaction had a statistically significant (*p* > 0.05) effect on free amine nitrogen (FAN) or protein carbonyl content (Table 4). These results indicate that proteolytic processes and protein oxidation were not affected by the experimental conditions.

### 3.5. Effect of Dry Sea Buckthorn Extract on Total Phenolic Content and Antioxidant Activity of the Traditional Kazakh Chunked Horse Meat Delicacy Jaya

The total phenolic content (TPC) and antioxidant activity of the two Jaya samples during storage at 0–4 °C are summarised in Table 5.

The addition of 3% sea buckthorn extract to the Jaya chunked delicacy resulted in a significant (*p* < 0.05) increase in total phenolic content (TPC) by 18.5%, in radical scavenging activity against DPPH by 9.6%, and in ferric (Fe^3+^) ion-chelating activity (FRAP) by 14.9% (Table 5).

### 3.6. Effect of Dry Sea Buckthorn Extract on the Microbiological Status of the Traditional Kazakh Chunked Horse Meat Delicacy Jaya

The microbiological status of the two Jaya samples during storage at 0–4 °C is presented in Table 6. Mesophilic aerobic and facultative anaerobic microorganisms, coliforms in 1.0 g of product, and *Salmonella* spp. in 25 g of product were not detected throughout the 30-day storage period in either the control or experimental samples (Table 6).

Only moulds, yeasts, and other spore-forming microorganisms were detected, with levels reaching up to 1 × 10^3^ CFU/g. These values remain within the permissible limits for cooked and smoked meat products. These results demonstrate that the applied processing technology for Jaya ensures the microbiological safety of the product, irrespective of the addition of 3% sea buckthorn extract.

## 4. Discussion

The results obtained concerning the instrumental colour changes on the cut surface of the control Jaya samples (Table 2) can be attributed to a series of chemical transformations that myoglobin undergoes during technological processing and storage, which influence colour development. Horsemeat is characterised by a rich raspberry-red hue due to its high heme iron content, primarily derived from myoglobin [32]. During the injection and tumbling processes, myoglobin interacts with sodium nitrite from the brine, forming metmyoglobin. Subsequently, prior to heat treatment, it reacts with nitric oxide—released from sodium nitrite through the action of denitrifying bacteria—resulting in the formation of nitrosomyoglobin [42].

Upon thermal processing of Jaya under mildly acidic conditions (pH 6.10; Figure 7), the globin component of nitrosomyoglobin denatures, yielding nitrosyl-hemochrome, a pinkish-red pigment responsible for the typical colour of cooked-smoked meat products [41]. When the cut surface is exposed to atmospheric oxygen and light, this pigment undergoes oxidation, leading to a fading of colour and a shift towards grey tones [42].

Jaya is subjected to intensive hot smoking using dense smoke. The phenolic compounds present in the smoke possess notable antioxidant properties. However, the penetration capacity of smoke is limited, with components accumulating only on the surface and within a few millimetres below it. This results in a caramel-brown coloration of the product’s outer surface, which is not solely due to smoke deposition but also to the Maillard reaction between protein degradation products and oxidised lipids. The resulting melanoidins, in conjunction with smoke components, contribute to the characteristic brown-red hue of the surface.

In the experimental samples, a slightly lower pH was observed, ranging between 5.77 and 5.97 (Figure 7), attributable to the acidic nature (pH 3.88) of the 3% dry sea buckthorn extract added to the brine. Sea buckthorn varieties generally exhibit pH values between 2.73 and 3.00 [27].

It is well established that the visual perception of red diminishes with decreasing pH, primarily due to the behaviour of myoglobin [42]. Therefore, the nitrosyl-hemochrome formed in a more acidic environment (pH 5.77) following heat treatment manifests in lighter tones and is more prone to oxidative fading [42]. The total phenolic content (TPC) of 42 mg GAE/g dry extract does not appear to be sufficient to inhibit this oxidation under the given acidic conditions. Similar colour degradation has been reported in pork fillet and cooked sausages injected with 1% and 3% sea buckthorn oil or extract (*Hippophae rhamnoides* L.) [6,31].

These observations are consistent with the pH changes in the brine (Figure 7) and the alterations in textural parameters of the final product. The addition of 3% sea buckthorn extract resulted in a statistically significant (*p* < 0.05) reduction in hardness, springiness, gumminess, and chewiness. In contrast, Anchidin et al. [6] reported a less pronounced pH decrease (to 6.17–6.19) in pork fillets injected with comparable concentrations of sea buckthorn oil.

The texture of meat products is largely influenced by the structural integrity of muscle proteins. In more acidic conditions, the water-holding capacity of these proteins is reduced, which alters their structural and mechanical properties [32]. As a result, the meat loses some of its retained moisture, which likely accounts for the observed decrease in stickiness, the firmer and rubberier texture, and the increased chewing difficulty of the experimental samples compared to the controls.

The slightly more acidic pH of the experimental Jaya samples may also explain the observed increase of 0.26 mg KOH/g in acid value (AV) and 0.12 mg Ala/g in free amino nitrogen (FAN) after 30 days of refrigerated storage in vacuum packaging. It is plausible that this lower pH promotes more active lipolytic and proteolytic reactions in the thermally processed Jaya.

The increase in TPC by 556.4 mg GAE/kg contributed to a 3.94% greater inhibition of DPPH radical activity and an increase of 779.64 mmol TE/kg in FRAP, indicating enhanced oxidative stability in samples containing sea buckthorn. This may explain the reduced formation of primary lipid oxidation products (as evidenced by a 0.10 meqO_2_/kg lower peroxide value (POV) and secondary oxidation products (0.13 mg MDA/kg lower TBARS levels) after 30 days of cold storage (0–4 °C).

This interpretation is further supported by the known presence of bioactive compounds in sea buckthorn (*Hippophae rhamnoides*), including fatty acids (e.g., ascorbic, malic, citric, and succinic acids, as well as polyunsaturated fatty acids), tocopherols, carotenoids, flavonoids, phytosterols, and other polyphenols [7,8,9,30,62].

Importantly, our findings indicate that protein oxidation was negligible in all samples. This suggests that the technological interventions applied—particularly smoking, vacuum packaging, and storage at low temperatures (0–4 °C)—were sufficient to inhibit protein oxidation within the product matrix. Consequently, any potential effect of the 3% sea buckthorn extract on protein oxidative stability was not detectable.

A comparable observation can be made regarding the microbiological condition of the two Jaia samples. The results indicate that the production technology employed for this equine delicacy—comprising hot air drying, boiling, and hot smoking, followed by rapid cooling, vacuum packaging, and storage at low temperatures (0–4 °C)—is sufficiently robust to ensure the microbial safety of the final product. In this context, the bactericidal effect attributed to the addition of 3% sea buckthorn extract to the brine used for injecting the horse meat appears to be largely overshadowed by the subsequent heat treatment, smoking, and other technological processes, making it difficult to assess its independent influence on the microflora. Nevertheless, it was observed that no mould or yeast spores were detected in the experimental Jaia samples after 30 days of refrigerated storage.

## 5. Conclusions

The obtained results and their evaluation partially confirmed the working hypothesis of this study, namely that the incorporation of 3.0% powdered water–ethanol sea buckthorn pomace extract into the injection brine of the traditional Kazakh chunked horse-meat delicacy Jaya can contribute to the stabilisation of the product’s quality and safety.

An enhancement in the antioxidant stability of Jaya was observed, accompanied by a moderately pronounced inhibitory effect on both hydrolytic and oxidative processes. Concurrently, a slight decrease in pH and a modification of the texture profile were recorded, along with noticeable discolouration on the cut surface of the product.

It is therefore recommended that further experiments be conducted using concentrations of powdered sea buckthorn pomace extract lower than 3.0% in the injection brine of the traditional Kazakh horse-meat delicacy Jaya, in order to optimise its technological and sensory properties.

## Figures and Tables

**Table 1:** Yield of dry sea buckthorn extract depending on ethanol concentration and extractant ratio.

Ethanol Concentration	Ratio	Output, g/40 g Liquid Extract	Output, %
50%	1:5	5.2	13.00
70%	1:5	7.2	18.00
90%	1:5	3.6	9.00
50%	1:10	3.9	9.75
70%	1:10	2.0	5.00
90%	1:10	1.3	3.25
50%	1:15	2.0	5.00
70%	1:15	1.9	4.75
90%	1:15	1.1	2.75

**Table 2:** Instrumental colour (L*, a*, b*) of samples during storage at 0–+4 °C.

Parameter	Control Sample		Experimental Sample	
L* *-1d*	50.56 ^bx^ ± 0.20		43.43 ^dy^ ± 0.17	
L* *-30d*	48.70 ^cy^ ± 0.28		53.07 ^ax^ ± 0.24	
*p-value*	*Factor* *Time of storage*	*Factor Addition of* *sea buckthorn extract*		*Factor* *interaction*
	*<0.001*	*<0.001*		*<0.001*
a* *-1d*	20.77 ^ax^ ± 0.30		20.10 ^bx^ ± 0.23	
a* *-30d*	20.84 ^ax^ ± 0.26		16.09 ^cy^ ± 0.17	
*p-value*	*Factor* *Time of storage*	*Factor Addition of* *sea buckthorn extract*		*Factor* *interaction*
	*<0.001*	*<0.001*		*<0.001*
b* *-1d*	7.29 ^bx^ ± 0.18		7.35 ^bx^ ± 0.20	
b* *-30d*	6.59 ^cy^ ± 0.20		9.32 ^ax^ ± 0.14	
*p-value*	*Factor* *Time of storage*	*Factor Addition of* *sea buckthorn extract*		*Factor* *interaction*
	*<0.001*	*<0.001*		*<0.001*

The results are presented as mean ± SEM; a, b, c, d, x, y index statistically significant (*p* ≤ 0.05) differences.

**Table 3:** Texture profile (TPA) of samples during storage at 0–4 °C.

Parameter	Control Sample	Experimental Sample	
Hardness, N-1d	55.43 ^b^ ± 0.56	39.17 ^c^ ± 0.61	
Hardness, N-30d	60.99 ^a^ ± 0.81	26.07 ^d^ ± 0.83	
*p-value*	*Factor* *Time of storage*	*Factor Addition of* *sea buckthorn extract*	*Factor* *interaction*
	*0.296*	*<0.001*	*0.025*
Cohesiveness-1d	0.57 ^ax^ ± 0.05	0.47 ^ax^ ± 0.08	
Cohesiveness-30d	0.48 ^bx^ ± 0.05	0.34 ^bx^ ± 0.06	
*p-value*	*Factor* *Time of storage*	*Factor Addition of* *sea buckthorn extract*	*Factor* *interaction*
	*40.00*	*0.003*	*0.436*
Springiness-1d	0.66 ^ax^ ± 0.03	0.55 ^ax^ ± 0.09	
Springiness-30d	0.57 ^ax^ ± 0.07	0.47 ^bx^ ± 0.07	
*p-value*	*Factor* *Time of storage*	*Factor Addition of* *sea buckthorn extract*	*Factor* *interaction*
	*0.040*	*0.017*	*0.914*
Gumminess, N-1d	33.22 ^a^ ± 0.37	23.53 ^b^ ± 0.63	
Gumminess, N-30d	32.43 ^a^ ± 0.68	10.05 ^c^ ± 0.46	
*p-value*	*Factor* *Time of storage*	*Factor Addition of* *sea buckthorn extract*	*Interactor* *interaction*
	*0.010*	*<0.001*	*0.017*
Chewiness, N * cm 1d	22.90 ^ax^ ± 0.31	16.75 ^bx^ ± 0.68	
Chewiness, N * cm 30d	20.65 ^ax^ ± 0.67	5.41 ^by^ ± 0.30	
*p-value*	*Factor* *Time of storage*	*Factor Addition of* *sea buckthorn extract*	*Factor* *interaction*
	*0.012*	*<0.001*	*0.062*
Resilience-1d	0.20 ^ax^ ± 0.03	0.20 ^ax^ ± 0.08	
Resilience-30d	0.15 ^ay^ ± 0.02	0.10 ^ay^ ± 0.02	
*p-value*	*Factor* *Time of storage*	*Factor Addition of* *sea buckthorn extract*	*Factor* *interaction*
	*0.003*	*0.238*	*0.306*

The results are presented as mean ± SEM; a, b, c, d, x, y index statistically significant (*p* ≤ 0.05) differences.

**Table 4:** Hydrolytic and oxidative changes in lipid and protein fractions during storage at 0–4 °C.

Parameter	Control Sample	Experimental Sample	
AV, mg KOH/g-1d	0.28 ^b^ ± 0.07	0.35 ^b^ ± 0.05	
AV, mg KOH/g-30d	0.33 ^b^ ± 0.04	0.59 ^a^ ± 0.09	
*p-value*	*Factor* *Time of storage*	*Factor Addition of* *sea buckthorn extract*	*Factor* *interaction*
	*0.002*	*<0.001*	*0.018*
POV, meqO_2_/kg-1d	0.11 ^b^ ± 0.02	0.11 ^b^ ± 0.02	
POV, meqO_2_/kg–30d	0.55 ^a^ ± 0.03	0.45 ^a^ ± 0.05	
*p-value*	*Factor* *Time of storage*	*Factor Addition of* *sea buckthorn extract*	*Factor* *interaction*
	*<0.001*	*0.063*	*0.047*
TBARS, mg MDA/kg-1d	0.47 ^ax^ ± 0.04	0.26 ^bx^ ± 0.02	
TBARS, mg MDA/kg-30d	0.49 ^ax^ ± 0.06	0.36 ^bx^ ± 0.02	
*p-value*	*Factor* *Time of storage*	*Factor Addition of* *sea buckthorn extract*	*Factor* *interaction*
	*0.057*	*<0.001*	*0.248*
FAN, mg Ala/g-1d	1.02 ± 0.19	1.14 ± 0.14	
FAN, mg Ala/g-30d	1.09 ± 0.14	1.21 ± 0.09	
*p-value*	*Factor* *Time of storage*	*Factor Addition of* *sea buckthorn extract*	*Factor* *interaction*
	*0.631*	*0.444*	*0.983*
Protein carbonyls, nmol DNPH/g protein-1d	<LoD	<LoD	
Protein carbonyls, nmol DNPH/g protein-30d	<LoD	<LoD	
*p-value*	*Factor* *Time of storage*	*Factor Addition of* *sea buckthorn extract*	*Factor* *interaction*
	*-*	*-*	*-*

The results are presented as mean ± SEM; a, b, x, index statistically significant (*p* ≤ 0.05) differences; LoD—Limit of detection.

**Table 5:** Total phenolic content and antioxidant activity during storage at 0–+4 °C.

Parameter	Control Sample	Experimental Sample	*p*-Value
TPC, mg GAE/kg	2458.22 ^b^ ± 13.00	3014.62 ^a^ ± 4.77	*0.0140*
% Inhibition of DPPH	37.09 ^b^ ± 1.31	41.03 ^a^ ± 1.05	*0.0329*
FRAP, mmol TE/kg	4456.30 ^b^ ± 17.80	5235.94 ^a^ ± 6.36	*0.0231*

The results are presented as mean ± SEM; a, b index statistically significant (*p* ≤ 0.05) differences.

**Table 6:** Microbiological status of chunked delicacy “Jaya” stored at 0–4 °C.

Samples	Time of Storage at 0–+4 °C			
	Day 5	Day 10	Day 15	Day 30
	Total aerobic plate count (TAPC), CFU/g			
Control sample	Not detected	Not detected	Not detected	Not detected
Experimental sample	Not detected	Not detected	Not detected	Not detected
	Moulds, yeasts, and other spore-forming microorganisms, CFU/g			
Control sample	Not detected	Not detected	1 × 10^2^	1 × 10^3^
Experimental sample	Not detected	Not detected	Not detected	Not detected
	Presence of coliforms in 1.0 g of product, CFU/g			
Control sample	Not detected	Not detected	Not detected	Not detected
Experimental sample	Not detected	Not detected	Not detected	Not detected
	Presence of *Salmonella* spp. in 25 g of product, CFU/g			
Control sample	Not detected	Not detected	Not detected	Not detected
Experimental sample	Not detected	Not detected	Not detected	Not detected

Total aerobic plate count (TAPC), an indicator of overall bacterial contamination, representing the number of mesophilic aerobic and facultative anaerobic microorganisms.

## Data Availability

The original contributions presented in this study are included in the article. Further inquiries can be directed to the corresponding authors.

## Abbreviations

ANOVA	Analysis of variance
AV	Acid value
DNPH	2,4-Dinitrophenylhydrazine
DPPH	2,2-Diphenyl-1-picrylhydrazyl
FAN	Free amino nitrogen
FRAP	The transition metals-chelating activity against ferric (Fe^3+^) ions—FRAP assay
GA	Gallic acid
GOST R	State Standard of Russia
LoD	Limit of detection
MDA	Malondialdehyde
POV	Peroxide value
RSM	Reflection surface method
SEM	Standard error of mean
TAPC	Total aerobic plate count
TBARS	Thiobarbituric acid reactive substances
TPA	Texture profile
TPC	Total phenolic content

## References

[1] Batyrbek A., Chisbiyah L.A. (2025). Exploring the role of traditional food in developing tourism in Kazakhstan. J. Tour. Culin. Entrep. (JTCE).

[2] Tayeva A., Kozhakhiyeva M., Jetpisbayeva B., Tlevlessova D., Samadun A., Valiyv A. (2023). Development of technology of boiled sausage from non-traditional raw materials. East.-Eur. J. Enter. Technol..

[3] Dias S., Castanheira E.M., Fortes A.G., Pereira D.M., Rodrigues A.R.O., Pereira R., Gonçalves M.S.T. (2020). Application of natural pigments in ordinary cooked ham. Molecules.

[4] Lohita B., Srijaya M.M., Chakraborty R., Mathur P., Roy S. (2024). Novel Technologies for Shelf-Life Extension of Food Products as a Competitive Advantage: A Review. Food Production, Diversity, and Safety Under Climate Change.

[5] Jaworska D., Sadowka A. (2024). Strategies to improve the functional value of meat and meat products. Foods.

[6] Anchidin B.G., Manoliu D.R., Ciobotaru M.C., Ciobanu M.M., Gucianu I., Sandu G.A., Boișteanu P.C. (2023). Development of a functional meat product with sea buckthorn oil and analysis of its sensory and physicochemical quality. Sci. Papers Ser. D Anim. Sci..

[7] Tereshchuk L.V., Starovoitova K.V., Vyushinsky P.A., Zagorodnikov K.A. (2022). The use of sea buckthorn processing products in the creation of a functional biologically active food emulsion. Foods.

[8] Tian J., Fang H., Chen S., Wei C., Wei X. (2025). Sea Buckthorn: A Functional Food Resource.

[9] Teleszko M., Wojdyło A., Rudzinska M., Oszmianski J., Golis T. (2015). Analysis of lipophilic and hydrophilic bioactive compounds content in sea buckthorn (*Hippophae rhamnoides* L.) berries. J. Agric. Food Chem..

[10] Wang Z., Zhao F., Wei P., Chai X., Hou G., Meng Q. (2022). Phytochemistry, health benefits, and food applications of sea buckthorn (*Hippophae rhamnoides* L.): A comprehensive review. Front. Nutr..

[11] Wang K., Xu Z., Liao X. (2022). Bioactive compounds, health benefits and functional food products of sea buckthorn: A review. Crit. Rev. Food Sci. Nutr..

[12] Ivanišová E., Blašková M., Terentjeva M., Grygorieva O., Vergun O., Brindza J. (2020). Biological properties of sea buckthorn (*Hippophae rhamnoides* L.) derived products. Acta Sci. Pol. Technol. Alim..

[13] Christaki E. (2012). *Hippophae rhamnoides* L. (Sea Buckthorn): A potential source of nutraceuticals. Food Public Health.

[14] Suryakumar G., Gupta A. (2011). Medicinal and therapeutic potential of sea buckthorn (*Hippophae rhamnoides* L.). J. Ethnopharm..

[15] Rafalska A., Abramowicz K., Krauze M. (2017). Sea buckthorn (*Hippophae rhamnoides* L.) as a plant for universal application. World Sci. News.

[16] Bayır H., Şimşek B.İ., Bayır Y. (2024). *Hippophae rhamnoides* L. botanical, medicinal, traditional, and current use of plant and fruits: A Review. New Trends Med. Sci..

[17] Ahani H., Attaran S. (2022). Therapeutic potential of Seabuckthorn (*Hippophae rhamnoides* L.) in medical sciences. Cell. Mol. Biomed. Rep..

[18] Tanwar H., Shweta, Singh D., Singh S.B., Ganju L. (2018). Anti-inflammatory activity of the functional groups presents in *Hippophae rhamnoides* (Sea buckthorn) leaf extract. Inflammopharmacology.

[19] Rédei D., Kúsz N., Jedlinszki N., Blazsó G., Zupkó I., Hohmann J. (2018). Bioactivity-guided investigation of the anti-inflammatory activity of *Hippophae rhamnoides* fruits. Planta Med..

[20] Zhu Y., Wu M., Li X., Wang Y., Li M., Zhou H. (2023). Flash extraction, characterization, and immunoenhancement activity of polysaccharide from *Hippophae rhamnoides* Linn. Chem. Biodiv..

[21] Dvorska D., Sebova D., Kajo K., Kapinova A., Svajdlenka E., Goga M., Frenak R., Treml J., Mersakova S., Strnadel J. (2025). Chemopreventive and therapeutic effects of *Hippophae rhamnoides* L. fruit peels evaluated in preclinical models of breast carcinoma. Front. Pharm..

[22] Süleyman H., Demirezer L.Ö., Büyükokuroglu M.E., Akcay M.F., Gepdiremen A., Banoglu Z.N., Göçer F. (2001). Antiulcerogenic effect of *Hippophae rhamnoides* L.. Phytother. Res..

[23] Pang X., Zhao J., Zhang W., Zhuang X., Wang J., Xu R., Xu Z., Qu W. (2008). Antihypertensive effect of total flavones extracted from seed residues of *Hippophae rhamnoides* L. in sucrose-fed rats. J. Ethnopharm..

[24] Park K.H., Hong J.H., Kim S.H., Kim J.C., Kim K.H., Park K.M. (2022). Anti-osteoporosis effects of the fruit of sea buckthorn (*Hippophae rhamnoides*) through promotion of osteogenic differentiation in ovariectomized mice. Nutrients.

[25] Jaśniewska A., Diowksz A. (2021). Wide spectrum of active compounds in sea buckthorn (*Hippophae rhamnoides*) for disease prevention and food production. Antioxidants.

[26] Muzykiewicz A., Zielonka-Brzezicka J., Klimowicz A. (2018). Antioxidant potential of *Hippophae rhamnoides* L. extracts obtained with green extraction technique. Herba Polon..

[27] Netreba N., Sandulachi E., Macari A., Popa S., Ribintev I., Sandu I., Boestean O., Dianu I. (2024). A study on the fruiting and correlation between the chemical indicators and antimicrobial properties of *Hippophae rhamnoides* L. *Horticulturae*
**2024**, *10*, 137. Horticulturae.

[28] Michel T., Destandau E., Le Floch G., Lucchesi M.E., Elfakir C. (2012). Antimicrobial, antioxidant and phytochemical investigations of sea buckthorn (*Hippophaë rhamnoides* L.) leaf, stem, root and seed. Food Chem..

[29] Buyukokuroglu M.E., Gulcin I. (2009). In vitro antioxidant and antiradical properties of *Hippophae rhamnoides* L.. Pharm. Mag..

[30] Papuc C., Nicorescu V., Crivineanu D.C., Goran G. (2009). Phytochemical constituents and free radicals scavenging activity of extracts from sea buckthorn fruits (*Hippophae rhamnoides*). Acta Hortic..

[31] Salejda A.M., Nawirska-Olszańska A., Janiewicz U., Krasnowska G. (2017). Effects on quality properties of pork sausages enriched with sea buckthorn (*Hippophae rhamnoides* L.). J. Food Qual..

[32] Kozhakhiyeva M., Dragoev S., Uzakov Y., Nurgazezova A. (2018). Improving of the oxidative stability and quality of new functional horse meat delicacy enriched with sea buckthorn (*Hippophae rhamnoides*) fruit powder extracts or seed kernel pumpkin (*Cucurbita pero* L.) flour. Com. Ren. l’Acad. Bulg. Sci..

[33] Wojtaszek A., Salejda A.M., Nawirska-Olszańska A., Zambrowicz A., Szmaja A., Ambrozik-Haba J. (2024). Physicochemical, antioxidant, organoleptic, and anti-diabetic properties of innovative beef burgers enriched with juices of açaí (*Euterpe oleracea* Mart.) and sea buckthorn (*Hippophae rhamnoides* L.) berries. Foods.

[34] Guo Z., Han L., Yu Q.L., Lin L. (2020). Effect of a sea buckthorn pomace extract-esterified potato starch film on the quality and spoilage bacteria of beef jerky sold in supermarket. Food Chem..

[35] Guo Z., Ge X., Gou Q., Yang L., Han M., Han G., Yu Q.-L., Han L. (2021). Changes in chilled beef packaged in starch film containing sea buckthorn pomace extract and quality changes in the film during super-chilled storage. Meat Sci..

[36] Park M.G., Jo S.Y. (2021). Comparison of biological activities of extracts from different parts of sea buckthorn (*Hippophae rhamnoides* L.). Korean J. Food Sci. Technol..

[37] Korekar G., Stobdan T., Singh H., Chaurasia O., Singh S. (2011). Phenolic content and antioxidant capacity of various solvent extracts from sea buckthorn (*Hippophae rhamnoides* L.) fruit pulp, seeds, leaves and stem bark. Acta Alim..

[38] He N., Wang Q., Huang H., Chen J., Wu G., Zhu M., Shao F., Yan Z., Sang Z., Cao L. (2023). A comprehensive review on extraction, structure, detection, bioactivity, and metabolism of flavonoids from sea buckthorn (*Hippophae rhamnoides* L.). J. Food Biochem..

[39] Sharma U.K., Sharma K., Sharma N., Sharma A., Singh H.P., Sinha A.K. (2008). Microwave-assisted efficient extraction of different parts of *Hippophae rhamnoides* for the comparative evaluation of antioxidant activity and quantification of its phenolic constituents by reverse-phase high-performance liquid chromatography (RP-HPLC). J. Agric. Food Chem..

[40] Wagh R.V., Chatli M.K. (2017). Response surface optimization of extraction protocols to obtain phenolic rich antioxidant from sea buckthorn and their potential application into model meat system. J. Food Sci. Technol..

[41] Meilgaard M.C., Carr B.T., Civille G.V. (1999). Sensory Evaluation Techniques.

[42] King D.A., Hunt M.C., Barbut S., Claus J.R., Cornforth D.P., Joseph P., Kim Y.H.B., Lindahl G., Mancini R.A., Nair M.N. (2023). American Meat Science Association guidelines for meat colour measurement. Meat Mus. Biol..

[43] Kolev N., Balev D., Dragoev S., Popova T., Petkov E., Dimov K., Suman S., Salim A.P., Vlahova-Vangelova D. (2025). Male layer-type birds (Lohmann Brown Classic Hybrid) as a meat source for chicken pâtés. Appl. Sci..

[44] Kolev N.D., Vlahova-Vangelova D.B., Balev D.K., Dragoev S.G. (2022). Stabilization of oxidative processes in cooked sausages by optimization of incorporated biologically active substances. Carp. J. Food Sci. Technol..

[45] Khan A.W. (1962). Extraction and fractionation of proteins in fresh chicken muscle. J. Food Sci..

[46] (2020). Animal and Vegetable Fats and Oils—Determination of Acid Value and Acidity. Published (Edition 4, 2020).

[47] Shantha N.C., Decker E.A. (1994). Rapid, sensitive, iron-based spectrophotometric methods for determination of peroxide values of food lipids. J. AOAC Int..

[48] Botsoglou N.A., Fletouris D.J., Papageorgiou G.E., Vassilopoulos V.N., Mantis A.J., Trakatellis A.G. (1994). Rapid, sensitive, and specific thiobarbituric acid method for measuring lipid peroxidation in animal tissue, food, and feedstuff samples. J. Agric. Food Chem..

[49] Moraru Manea A.I., Cocan I., Dumbrava D.G., Poiana M.A. (2025). Effect of fruit powders as natural alternatives to sodium nitrite on lipid oxidation in clean-label salami. Foods.

[50] Vassilev K., Ivanov G., Balev D., Dobrev G. (2012). Protein changes of chicken light and dark muscles during chilled storage. J. EcoAgriTourism.

[51] Mercier Y., Gatellier P., Renerre M. (2004). Lipid and protein oxidation in vitro, and antioxidant potential in meat from Charolais cows finished on pasture or mixed diet. Meat Sci..

[52] Vardakas A., Kechagias A., Penov N., Giannakas A.E. (2024). Optimization of enzymatic-assisted extraction of bioactive compounds from *Olea europaea* leaves. Biomass.

[53] Brand-Williams W., Cuvelier M.E., Berset C.L.W.T. (1995). Use of a free radical method to evaluate antioxidant activity. LWT—Food Sci. Technol..

[54] Dinkova R., Heffels P., Shikov V., Weber F., Schieber A., Mihalev K. (2014). Effect of enzyme-assisted extraction on the chilled storage stability of bilberry (*Vaccinium myrtillus* L.) anthocyanins in skin extracts and freshly pressed juices. Food Res. Int..

[55] Benzie I.F., Strain J.J. (1996). The ferric reducing ability of plasma (FRAP) as a measure of “antioxidant power”: The FRAP assay. Anal. Biochem..

[56] Drake M.A., Watson M.E., Liu Y. (2023). Sensory analysis and consumer preference: Best practices Ann. Rev. Food Sci. Technol..

[57] Meat and Meat Products. General Requirements and Methods of Microbiological Testing.

[58] Food Products. Methods for Determination Quantity of Mesophilic Aerobes and Facultative Anaerobes.

[59] Food Products. Methods for Detection and Quantity Determination of Coliforms.

[60] Food Products. Methods for the Detection of *Salmonella* spp..

[61] Kolev N., Vlahova-Vangelova D., Balev D., Dragoev S. (2022). Quality changes of cooked sausages influenced by the incorporation of a three-component natural antioxidant blend. BIO Web Conf..

[62] Ma Q.-G., He N.-X., Huang H.-L., Fu X.-M., Zhang Z.-L., Shu J.-C., Wang Q.-Y., Chen J., Wu G., Zhu M.-N. (2023). *Hippophae rhamnoides* L.: A comprehensive review on the botany, traditional uses, phytonutrients, health benefits, quality markers, and applications. J. Agric. Food Chem..

